# Coastal Upwelling Supplies Oxygen-Depleted Water to the Columbia River Estuary

**DOI:** 10.1371/journal.pone.0018672

**Published:** 2011-04-20

**Authors:** G. Curtis Roegner, Joseph A. Needoba, António M. Baptista

**Affiliations:** 1 NOAA Fisheries, Northwest Fisheries Science Center, Point Adams Research Station, Hammond, Oregon, United States of America; 2 Science and Technology Center for Coastal Margin Observation and Prediction, Oregon Health and Science University, Beaverton, Oregon, United States of America; Universidade de Vigo, Spain

## Abstract

Low dissolved oxygen (DO) is a common feature of many estuarine and shallow-water environments, and is often attributed to anthropogenic nutrient enrichment from terrestrial-fluvial pathways. However, recent events in the U.S. Pacific Northwest have highlighted that wind-forced upwelling can cause naturally occurring low DO water to move onto the continental shelf, leading to mortalities of benthic fish and invertebrates. Coastal estuaries in the Pacific Northwest are strongly linked to ocean forcings, and here we report observations on the spatial and temporal patterns of oxygen concentration in the Columbia River estuary. Hydrographic measurements were made from transect (spatial survey) or anchor station (temporal survey) deployments over a variety of wind stresses and tidal states during the upwelling seasons of 2006 through 2008. During this period, biologically stressful levels of dissolved oxygen were observed to enter the Columbia River estuary from oceanic sources, with minimum values close to the hypoxic threshold of 2.0 mg L^−1^. Riverine water was consistently normoxic. Upwelling wind stress controlled the timing and magnitude of low DO events, while tidal-modulated estuarine circulation patterns influenced the spatial extent and duration of exposure to low DO water. Strong upwelling during neap tides produced the largest impact on the estuary. The observed oxygen concentrations likely had deleterious behavioral and physiological consequences for migrating juvenile salmon and benthic crabs. Based on a wind-forced supply mechanism, low DO events are probably common to the Columbia River and other regional estuaries and if conditions on the shelf deteriorate further, as observations and models predict, Pacific Northwest estuarine habitats could experience a decrease in environmental quality.

## Introduction

Low dissolved oxygen (DO) in aquatic and marine systems is an established and growing concern worldwide [1]. Many recent papers have reported increases in the occurrence and extent of areas affected by low DO events [2], and the deleterious effects of low DO are well-documented on scales ranging from individual organisms to entire ecosystems [3]. Oxygen-depleted conditions in estuarine and shallow marine areas are often attributed to anthropogenic nutrient enrichment delivered by terrestrial-fluvial pathways [4]. Subsequent phytoplankton production and eventual decomposition by heterotrophic bacterial consumption can reduce dissolved oxygen in bottom waters. However, low DO conditions also occur naturally in subsurface waters of the worlds' oceans [5], [6], and wind-forced upwelling can propel this water onto continental shelves and into shallow water bays and estuarine systems [7], [8].

Oxygen is a non-conservative tracer, as its concentration is affected by many factors, including photosynthesis and respiration, and exchange rates between surface waters and the atmosphere or bottom waters and the benthos. Dissolved oxygen levels can vary from supersaturated to anoxic (0% saturation), often over short (semidiurnal) temporal and narrow (meter) spatial scales (e.g., [9], [10]). Supersaturated conditions are usually caused by photosynthetic production during daylight. The critical concentration delimiting hypoxia is often defined at <2.0 mg L^−1^ (1.4 mL L^−1^; ∼30% atmospheric saturation) [2], [4]. While mass mortalities are a major consequence of hypoxic conditions, for many organisms the negative effects of low DO occur well above the 2.0 mg L^−1^ threshold [11]–[13]. Such sublethal effects include stress-related reductions in growth and reproduction, motility, feeding rates, and altered predator-prey relationships [11], [14], [15]. Bricker et al. [16] define “biological stress” as oxygen concentrations between 2.0 and 5.0 mg L^−1^. Breitburg et al. [3] define hypoxia “mechanistically as oxygen concentrations that are sufficiently reduced that they affect the growth, reproduction, or survival of exposed animals, or result in avoidance behaviors.” It has become clear that sublethal effects must be considered when assessing the impacts of low DO events on ecosystems.

The oceanography of Northeast Pacific coastal waters is strongly influenced by wind-forced upwelling dynamics [17], [18]. During upwelling, equatorward winds draw nutrient-rich, and often reduced DO, subsurface water into the photic zone, where phytoplankton growth is stimulated. Oceanographic observations have routinely detected reduced DO levels in deep water year-round, but also in shallower shelf waters during active upwelling [7], [19]–[21]. On occasion, these upwelled waters are severely hypoxic. Recently, hypoxia and mass mortalities of benthic invertebrates and fish were observed on the Oregon shelf, and these phenomena were attributed to both upwelling dynamics (the decay of phytoplankton blooms at depth) and changes in the character of subsurface circulation patterns [20]–[23]. However, low DO events in coastal areas of the Pacific Northwest are not new. Over forty years ago, Pearson and Holt [24] measured hypoxia (<1.5 mg L^−1^) in the nearshore ocean and within Grays Harbor, Washington, and Haertal et al. [25] documented near-hypoxic concentrations (2.2 mg L^−1^) in high salinity water in the Columbia River estuary. Both groups of researchers attributed these low DO events to upwelling dynamics.

Previous work in the Columbia estuary has established links between wind stress, estuarine salinity, and chlorophyll concentration [25], [26], and a strong wind-forced ocean-estuary connection has been found for many coastal estuaries in the Pacific Northwest. In Oregon these include Coos Bay [27], [28], Alsea Bay [29], Yaquina Bay [30], [31], and Tillamook Bay [32], and in Washington studies have been made in Willapa Bay [19], [28], [33], [34] and Grays Harbor [24], [28], [33]. Other work has revealed the influence of Columbia River outflows in delivering high concentrations of phytoplankton [26], [35] and nutrients [36], [37] to the estuary and nearshore ocean. These terrestrial-fluvial sources of organic matter and nutrients constitute a leading mechanism for generating oxygen stress in estuarine and coastal sites on a worldwide basis [2]. However, DO levels in the Columbia estuary have not been routinely assessed, and it is not clear whether stressful low oxygen conditions continue to exist in the system, and, if so, from whence they are derived. In this context, note that the Columbia estuary is a critical migration and rearing habitat for endangered juvenile and adult anadromous Pacific salmon (*Oncorhynchus* spp.), ecologically relevant forage fishes (smelt, anchova, herring), and the economically important Dungeness crab (*Cancer magister*). It is not known whether low DO events in the Columbia estuary are a problem for these organisms.

Here we convey the results of field studies designed to determine the source, forcing, and resultant spatial and temporal patterns of oxygen concentration in the Columbia estuary. Specifically, we

1. measured the variation in oxygen concentrations in relation to salinity to determine end-member advection versus in situ sources or sinks;
2. evaluated the effects of upwelling wind and tidal forcings on patterns of estuarine oxygen concentration;
3. determined the vertical and horizontal spatial extent of low DO intrusions; and
4. estimated interannual variation in the number of seasonal upwelling events and the cumulative impact of low DO events among the years 2006–2008.

We then discuss possible consequences of these DO observations on migrating juvenile salmon and the life stages of Dungeness crab based on information found in the literature.

## Methods

### Water quality measurements

**Figure 1:**
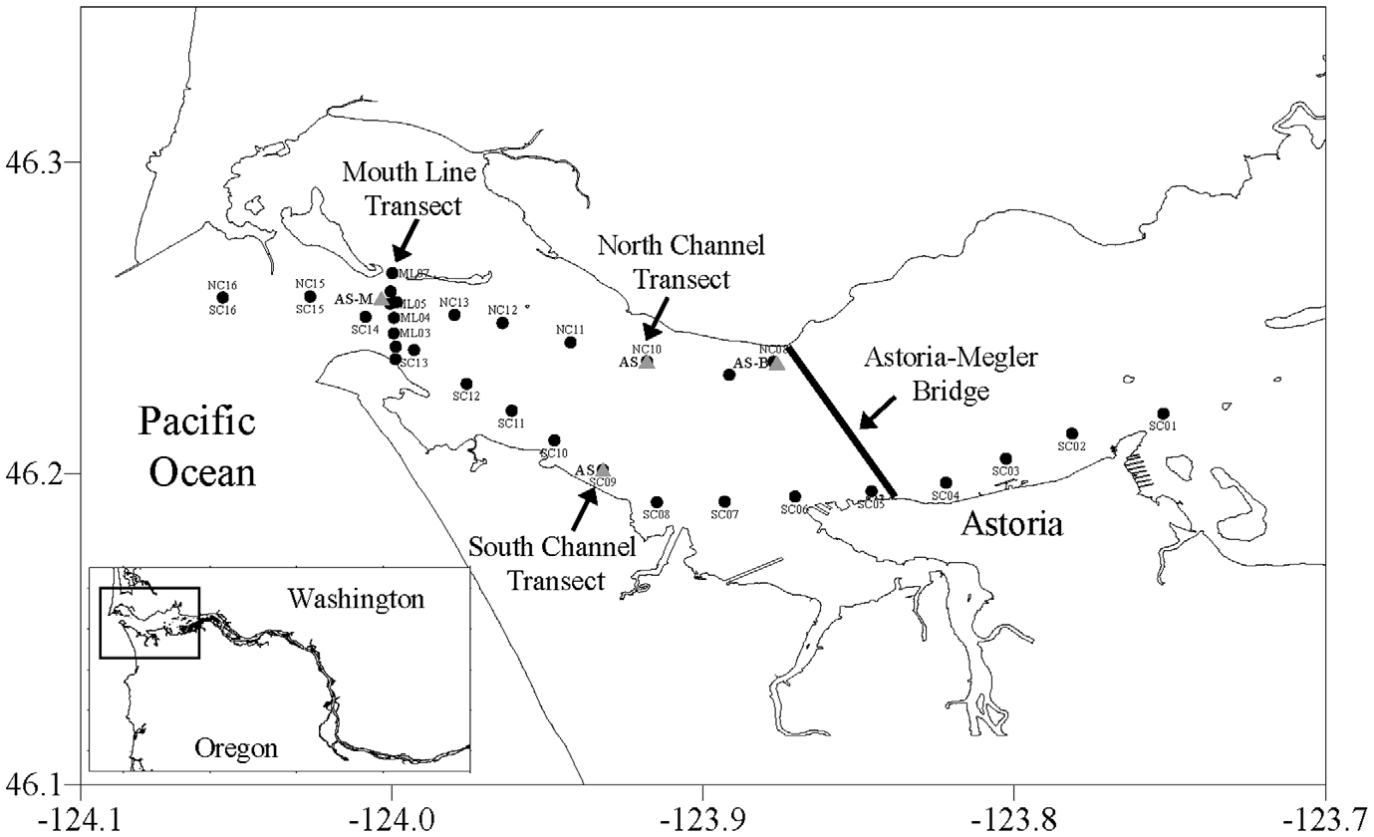
Columbia River estuary sample stations. Circles show transect stations; triangles indicate anchor stations. The inset identifies the regional location.

From 2006 through 2008, hydrographic measurements were made from transect (spatial survey) or anchor station (temporal survey) cruises (Figure 1). Transects included:

1. South Channel, composed of 16 stations along a 27 km transect of the Columbia River South Channel (shipping lane) extending from Buoy 10 (river kilometer (rkm) 0) to Tongue Point (rkm 27);
2. North Channel, with 9 stations on a 14 km transect from Buoy 10 to the Astoria-Melger Bridge (rkm 14); and
3. Mouth Line, composed of 8 stations on a 3.5 km north-south transect aligned near the mouth of the Columbia River between Clatsop Spit, OR, and Baker Bay, WA, at about rkm 5.

Observations along these three transects were made at various stages of the tide and spring/neap cycle (Table 1). Anchor stations for temporal studies were located at ∼20 m depth at station NC14, and at ∼15 m depth near stations NC07, NC10, and SC09 (Figure 1).

**Table 1:** Physical metrics and O_2_-S regression statistics during the anchor station (AS) and survey cruises in the Columbia River estuary during 2006–2008.

Cruise	CruiseID	Date	DOY	Type	LI	S/N	CWS	S_max_	O_2min_	Regression		
										m	r^2^	n
FR26	A	22Aug06	234	Survey	0.06	S	-0.20	32.7	4.8	-0.12	0.82	534
FR27	B	09Sep06	251	AS	1.00	S	-0.22	31.9	3.7	-0.11	0.42	722
FR28	C	13Sep06	256	Survey	0.73	N	-0.26	31.8	4.0	-0.11	0.61	1399
FR29	D	21Sep06	264	Survey	0.04	S	0.13	31.5	6.2	<0.01	0.02	1096
FR30	E	15Oct06	289	Survey	0.37	N	0.00	31.8	6.6	<0.01	0.03	1024
FR33	F	08May07	128	Survey	0.78	S	-0.01	31.7	6.8	-0.08	0.51	663
FR34	G	15May07	135	Survey	0.09	S	-0.33	32.8	4.3	-0.14	0.69	470
FR37	H	31May07	151	Survey	0.97	S	-0.33	32.0	4.8	-0.07	0.34	345
FR38	I	20Aug07	232	Survey	0.35	N	0.12	30.3	7.4	<0.01	0.02	1598
FR39	J	21Aug07	233	AS	0.44	N	0.07	29.7	7.6	-0.01	0.04	1411
FR40	K	22Aug07	234	AS	0.63	N	0.04	28.2	7.1	-0.03	0.34	345
FR41	L	27Aug07	239	Survey	0.98	S	-0.28	32.2	4.8	-0.08	0.67	1609
FR42	M	28Aug07	240	AS	1.00	S	-0.28	31.0	4.6	-0.11	0.79	1641
FR43	N	29Aug07	241	AS	0.99	S	-0.27	32.6	3.6	-0.09	0.53	1870
Barnes1	O	10Jul08	191	AS	0.32	N	-0.72	32.9	2.2	-0.19	0.92	223
Barnes2	P	13Jul08	194	AS	0.61	N	-0.61	33.2	2.1	-0.24	0.94	154
Barnes4	Q	18Jul08	199	AS	0.96	S	-0.26	32.3	2.6	-0.16	0.90	397
Barnes5	R	20Jul08	201	AS	1	S	-0.23	31.3	3.3	-0.14	0.91	411

DOY: day of year. LI: lunar index. S/N: spring/neap cycle. CWS: 4-d cumulative wind stress (N m^−2^; where negative denotes upwelling, positive is downwelling, and low wind stress ranges from 0.03 to -0.03 N m^−2^). S_max_: maximum observed salinity (psu). O_2min_: minimum observed oxygen concentration (mg L^−1^). m: slope of O_2min_-S_max_ regression line. r^2^: variation explained. n: number of observations. All regression equations were significant (*P*<0.001).

At each station, vertical profiles were made either with a Sea Bird Electronics (SBE) 19 plus Conductivity-Temperature-Depth (CTD) probe (2006 and 2007) or with a SBE 9/11 CTD (2008), each equipped with a SBE 43 dissolved oxygen sensor. Data were recorded at 2 Hz and binned into 0.5 m depth intervals (typically yielding >5 measurements per bin), and Surfer 8® (Golden Software) was used to interpolate salinity and oxygen profiles either spatially (transects) or temporally (anchor stations). Note that spatial interpolations were semisynoptic views biased by the speed of the research vessel. At anchor stations during 2006 and 2007, CTD casts were made at approximately 0.5 h intervals, while sampling periods were more irregular during 2008. The CTD data were used to compile oxygen-salinity (O_2_-S) scatterplots and to determine maximum salinity (S_max_) and minimum oxygen (O_2min_) values.

Since the effects of low DO on migrating salmonids was of particular concern, we used literature values to categorize concentrations determined to cause biological stress in salmon [11], [14], [38], [39]. Five categories of oxygen concentration were assigned:

1. hypoxic or severe biological stress (0 to 2 mg L^−1^);
2. moderate biological stress (>2 to 4 mg L^−1^);
3. mild biological stress (>4 to 6 mg L^−1^);
4. normoxic (>6 to ∼9 mg L^−1^); and
5. supersaturated (>9 or 10 mg L^−1^).

Categories 1–3 are regarded here as low DO conditions. Note that oxygen saturation levels varied by date (based primarily on oxygen solubility in relation to temperature), and 100% saturated levels were approximately 9 mg L^−1^ in summer-autumn 2006 and 2007, and 10 mg L^−1^ in May 2007. Oxygen saturation was not determined during 2008.

### Time series measurements of wind and tide

Variation in wind forcing affecting estuarine hydrology was assessed with time series of coastal wind stress. Wind velocity data was measured at the Columbia River Bar buoy (Station 46029; 46.12°N, 124.51°W; http://ndbc.noaa.gov), except during April–June 2007, when buoy loss necessitated use of data from Station 46041 (47.34°N 124.75°W). Wind vectors were converted to mean daily alongshore wind stress (τ_N_, N m^−2^) from hourly observations. Positive (northward) wind stress induces downwelling and negative (southward) wind stress drives upwelling along the Pacific Northwest coast. To provide an index of the strength of wind forcing in the days prior to hydrographic sampling, we calculated time series of the 4-d cumulative mean daily wind stress (CWS). This value was based on the 1–3 d lag of the best fit cross-correlations found between wind stress and maximum daily salinity in the estuary [26].

Water level data was acquired from Tongue Point NOAA tide station (123.7°N, 46.2°W). Tides are a mixed semidiurnal type in the Columbia estuary. The ebb-to-flood stage of tide (SOT) during sample times was designated by assigning 0 to low water and 1.0 to high water, and partitioning the time intervals between lows and highs. Periods of flooding water were assigned positive values and periods of ebbing water were assigned negative values (e.g., 0.5 corresponds to mid-flood tide while -0.5 is mid-ebb tide). These data were used to identify semidiurnal tidal conditions during transect and anchor station sampling. The spring/neap (S/N) cycle was also set to range from 0 to 1.0, where neap periods ranged from 0.25 to 0.75 and spring periods from <0.25 to 0 and >0.75 to 1.0. The S/N cycle strongly influences circulation patterns and water column structure in the Columbia estuary [26], [40].

### Analysis

For each survey date, we used linear regression of oxygen-salinity (O_2_-S) scatterplots to evaluate the source of oxygen to the estuary, where negative slopes indicate higher oxygen concentrations in the river end-member than the ocean end-member, and positive slopes indicate the converse [26], [27], [37]. Data from all types of transects or time series measurements were combined for each sample date.

To test for the influence of forcing functions on the distribution of estuarine water properties, we regressed daily wind (CWS) and tidal (S/N) indices with maximum daily salinity (S_max_) and minimum daily oxygen (O_2min_) values. We also plotted the slope of individual O_2_-S regressions (derived above) by CWS to examine the effect of wind stress on the estuarine oxygen gradient.

The spatial scale of exposure to low DO was estimated by integrating depth-distance contour plots of DO isopleths generated from transect surveys. We used the Mouth Line transect for cross-channel and the South Channel transect for along-channel determinations (Figure 1). Cross-channel transects were made at various stages of the tide and highlight tidal-scale variation of water quality parameters. For the along-channel transects, we concentrated on samples made near slack high water to evaluate the spatial extent of low DO events. We determined area (vertical × horizontal, m^2^) and percent of area surveyed (total area depended on transect length) for each of the DO categories described above. Dates of the surveys are given in Table 1 along with associated tidal, wind, and hydrographic data.

As a measure of the impact of low DO events at the benthic layer, we determined the length of exposure (h) of each DO category in bottom water using time series data from anchor station studies, which we expressed as the percent of the observation period. Due to strong differences in water column stratification between spring and neap tides, we also noted the maximum vertical extent of biologically stressful water during each measurement period.

For evaluation of interannual variation during 2006–2008, we estimated the number of low DO events and the duration (d) of low DO conditions in the estuary. The period 30 March–26 November (day of year 90 through 300) was chosen to encompass the typical upwelling period in the Pacific Northwest. The number of upwelling events each year was visually assessed from time series of northward wind stress, where an event is defined as a period of at least three continuous days of τ_N_ < −0.03 N m^−2^ [26]. To estimate the number of days experiencing low DO in the estuary, we applied the regression equation O_2min_  = 6.47× (CWS) +6.0 (derived above) to the CWS time series to yield the daily minimum O_2_ level. We then summed the number of days for each oxygen category described above. This simple model assumes the linear relation between DO and the strength of upwelling winds would hold for values outside those measured in the field.

## Results

Of the 18 cruises made from 2006 through 2008, nine occurred during spring tide and upwelling conditions, two during spring tide and downwelling or low wind stress, three during neap tide and upwelling, and four during neap tide and downwelling (Table 1).

**Figure 2:**
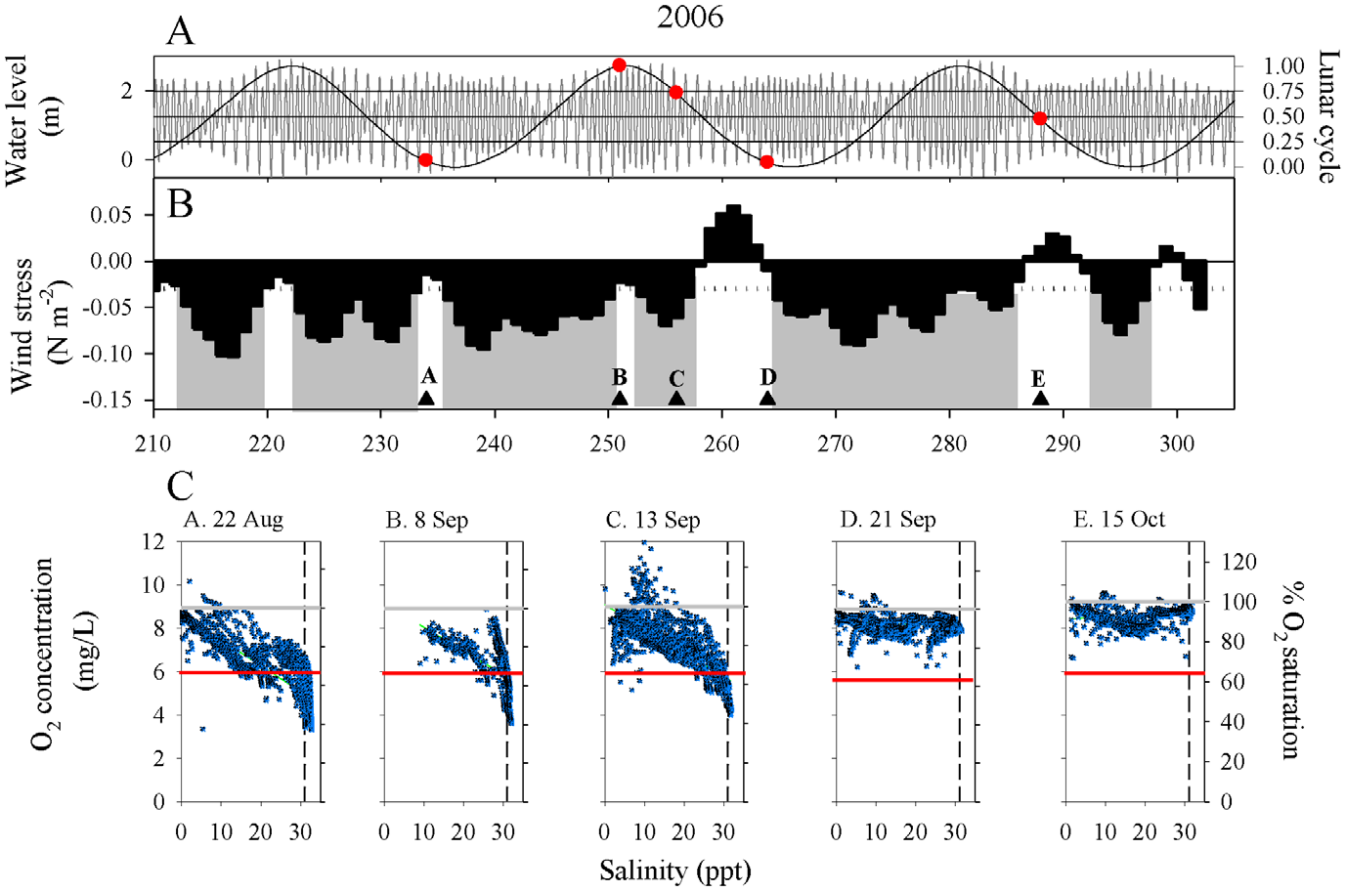
Time series of physical drivers and O_2_-S scatterplots during 29 July through 6 November 2006. A. Tidal (left axis) and spring-neap (right axis) cycles. Circles denote cruise dates. B. Bars: Low-pass filtered mean daily northward wind stress (τ_N_, N m^−2^). Gray shaded sections denote upwelling events defined as ≥3 consecutive days of τ_N_ < −0.03 (dotted line). Triangles and letters indicate the timing of hydrographic cruises. C. O_2_-S scatterplots for designated cruises. Oxygen values are expressed as both concentration (mg L^−1^) and as percent saturation. Red horizontal line is the 6 mg L^−1^ criterion. Grey horizontal line is the 100% saturation level. Black dashed vertical line denotes upwelled water at 31 psu. Regression statistics are in Table 1.

O_2_-S scatterplots were used to examine DO levels in ocean, estuarine, and river water masses during 2006–2008 (Figures 2–4). DO in the estuary was heterogeneous in space and time, due to both advection from ocean and river end-members, and also from in situ production. During the survey dates, DO ranged from supersaturated (11.7 mg L^−1^; 132% saturation) to near hypoxic (2.1 mg L^−1^; ∼30% saturation). Slopes of O_2_-S regressions varied among sample dates from strongly negative to near zero (Table 1). The river end-member was always normoxic (>6.0 mg L^−1^) and usually >90% saturated. In contrast, water imported during flood tide varied widely in both oxygen concentration and maximum salinity, with higher salinity water generally being reduced in DO. Supersaturated conditions occurred in estuarine water (3–20 psu) associated with high chlorophyll concentrations, particularly blooms of the “red water” ciliate *Myrionecta rubra* (see below).

**Figure 3 pone-0018672-g003:**
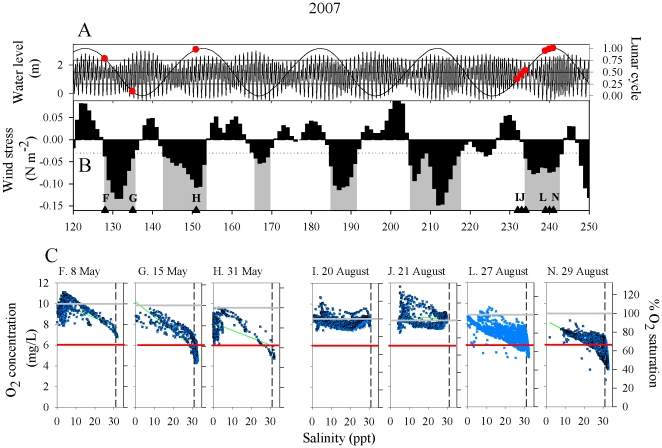
Time series of physical drivers and O_2_-S scatterplots 30 April through 7 September 2007. A. Tidal and spring-neap cycles. Circles denote cruise dates. B. Bars: Low-pass filtered mean daily northward wind stress (τ_N_, N m^−2^). Gray shaded sections denote upwelling events defined as ≥3 days of consecutive τ_N_ < −0.03 (dotted line). Triangles and letters indicate the timing of hydrographic cruises. C. O_2_-S scatterplots for designated cruises. Oxygen values are expressed as both concentration (mg L^−1^) and as percent saturation. Red horizontal line is the 6 mg L^−1^ criterion. Black dashed vertical line denotes upwelled water at 31 psu. Regression statistics are in Table 1.

**Figure 4:**
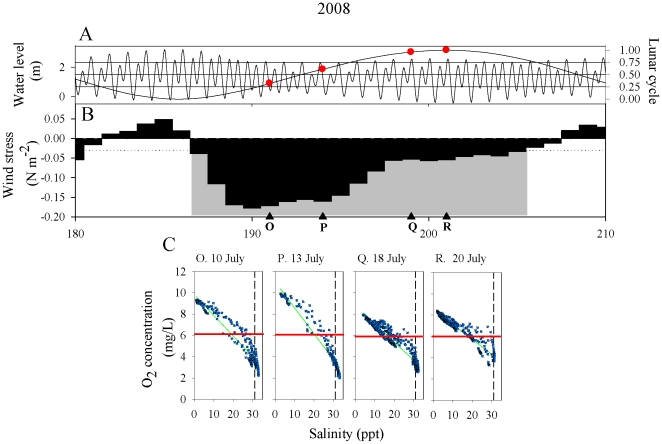
Time series of physical drivers and O_2_-S scatterplots 29 June through 29 July 2008. A. Tidal and spring-neap cycles. Circles denote cruise dates. B. Bars: Low-pass filtered mean daily northward wind stress (τ_N_, N m^−2^). Gray shaded sections denote upwelling events defined as ≥3 days of consecutive τ_N_ < −0.03 (dotted line). Triangles and letters indicate the timing of hydrographic cruises. C. O_2_-S scatterplots for designated cruises. Oxygen values are expressed as both concentration (mg L^−1^) and as percent saturation. Red horizontal line is the 6 mg L^−1^ criterion. Black dashed vertical line denotes upwelled water at 31 psu. Regression statistics are in Table 1.

In 2006 we made observations during late August through mid-October (Figure 2). Cruises A, B, and D were made during spring tides, C was transitional, and E was conducted during a neap tide. Cruises A–C occurred during a long period of upwelling; all O_2_-S regressions had negative slopes with moderate to weakly stressful DO levels in the ocean end-member. Cruises D and E occurred during downwelling conditions, regression equations had slopes near zero, and no low DO conditions were detected (concentrations at all salinities were normoxic). In 2007, we sampled in May and August (Figure 3). In May, Cruise F sampling occurred during the transition from downwelling to upwelling conditions (low wind stress), while Cruises G and H were conducted during upwelling. While all three O_2_-S regressions had negative slopes, only the cruises during upwelling had stressful (albeit weakly stressful) DO levels. In August we sampled during neap and spring tides. Neap tide samples (I–K) occurred during downwelling, and DO levels were supersaturated or normoxic across the salinity range. Spring tide cruises (L–N) occurred during upwelling, slopes were negative, and reduced DO levels were found in the ocean end-member. In 2008, we sampled both neap and spring tides during a protracted upwelling period (Figure 4). All O_2_-S regression lines were negative with levels approaching the hypoxic threshold. Thus, upwelling conditions brought various levels of oxygen-depleted water to the estuary, while during downwelling conditions estuarine, waters in the estuary were consistently normoxic or supersaturated.

**Figure 5:**
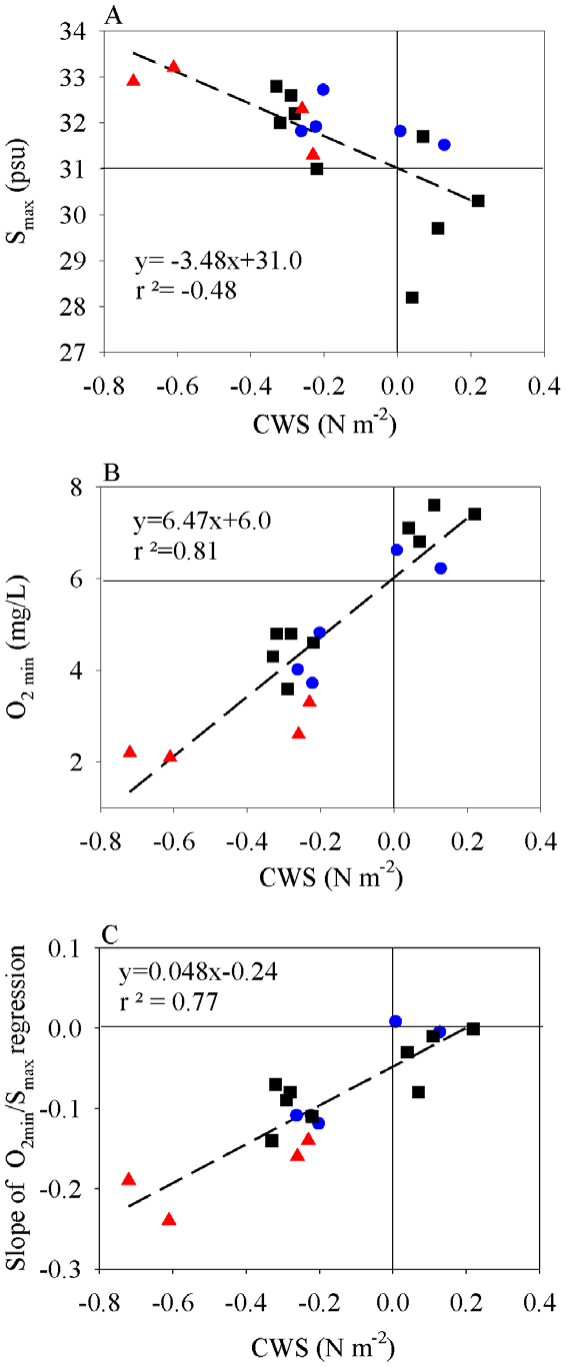
Scatter plots and regression statistics. A. Maximum salinity (S_max_) by cumulative northward 4-d wind stress (CWS); B. Minimum oxygen concentration (O_2min_) by cumulative 4-d wind stress; C. Regression coefficients from O_2min_-S_max_ regression by cumulative 4-d wind stress. Black = 2006; blue = 2007; red = 2008.

Alongshore wind stress was a main driver for variation in salt and oxygen concentrations imported into the estuary. S_max_ was negatively related to the 4-d cumulative wind stress CWS (*P* = 0.014; r^2^ = −0.48; Figure 5A), while O_2min_ was positively related to CWS (*P*<0.001; r^2^ = 0.81; Figure 5B). O_2min_ was also negatively related to S_max_ (*P*<0.001; r^2^ = −0.51; Figure not shown). These results corroborate that during upwelling favorable periods, low DO levels occurred with high salinity water (usually >29 psu); during downwelling periods DO concentrations were usually normoxic (Figures 2–4). Additionally, the O_2min_-S_max_ regression slopes were positively related to CWS (*P*<0.001; r^2^ = 0.77; Figure 5C), indicating an increasing input of high salinity, low DO water with increasing upwelling wind stress (Table 1; Figure 5C). Conversely, slopes were weakly negative or near zero during downwelling. Neither S_max_ nor O_2min_ were significantly related to the spring-neap index (*P* = 0.67 and 0.24, respectively). The oxygen concentration of ocean water advected into the Columbia estuary thus varied in relation to the direction and intensity of the alongshore winds.

**Figure 6:**
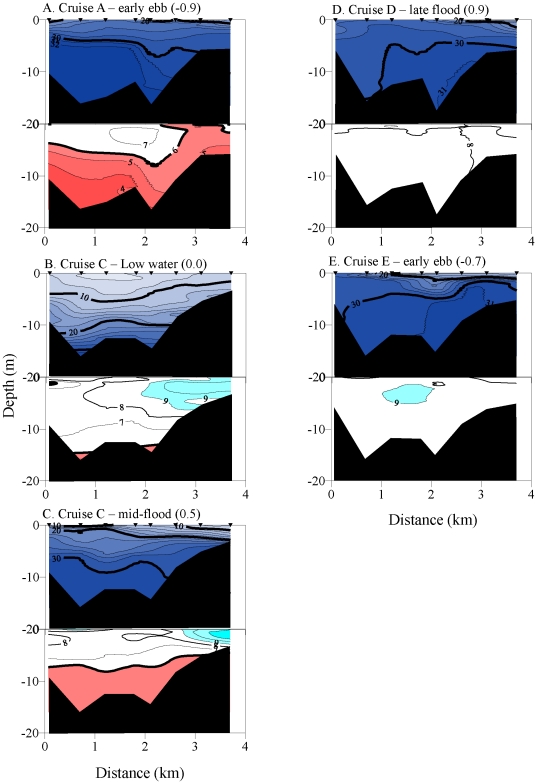
Across-channel transects of vertical water column salinity and dissolved oxygen concentration during Autumn 2006. Salinity, top panels. Oxygen concentration, bottom panels. Plots A–C occurred during upwelling conditions, and plots D and E during downwelling conditions. Plots A, D, and E occurred near high water, while Plots B and C occurred on the same date but different stages of the tide. Salinity isopleths are 2 psu. Oxygen isopleths are 1 mg L^−1^, Supersaturated = blue; normoxic = white; stressed =  red. Bottom contours are shown in black. Triangles designate location of measurements.

To evaluate the vertical and horizontal spatial extent of low DO water in the Columbia estuary, we integrated depth-distance contour plots of DO isopleths generated from transect surveys. Cross-channel transects at the Mouth Line showed that large percentages of the water column were affected by low DO water (Table 2). During Cruise A, 66.3% of the water column had reduced oxygen concentrations, including 16.3% of the transect at moderate biological stress levels (Figure 6A). Note the ventilation of low DO water to the surface. Two measurements of the Mouth Line transect were made during Cruise C, the first near low tide and the second near mid-flood (Figure 6B and C). During low tide, 10.1% of the water column was supersaturated at the surface, with small pools of low DO water at depth. By the subsequent transect (mid-flood), low DO water had filled the channel from the bottom to 5–6 m below the surface (42.3% of the area) while the supersaturated zone increased in intensity but decreased in area. Cruises D and E occurred during downwelling conditions, when oxygen concentrations were normoxic or supersaturated even at salinities >31 psu (Figure 6D and E).

**Table 2:** Interpolated area and percent of water column composed of each DO category during cross- and along channel surveys of the Columbia River estuary.

Cruise ID	Rep	Tide	Mouth Line transects					Percent of water column			
			Area of O_2_ category (10^3^ m^2^)								
			>2–4	>4–6	>6–10	>10	Σ <6	>2–4	>4–6	>6–10	>10
A	1	-0.90	1.0	3.0	2.0	0.0	4.0	16.3	50.0	33.7	0.0
C	1	0.00	0.0	0.2	4.9	0.6	0.2	0.0	2.8	87.1	10.1
	2	0.50	0.0	2.4	3.1	0.2	2.4	0.0	42.3	54.3	3.3
D	1	0.90	0.0	0.0	5.4	0.0	0.0	0.0	0.0	100.0	0.0
E	1	-0.33	0.0	0.0	5.5	0.0	0.0	0.0	0.0	100.0	0.0
F	1	-0.33	0.0	0.0	4.0	2.5	0.0	0.0	0.0	61.8	38.2
	2	0.33	0.0	0.0	3.9	1.8	0.0	0.0	0.0	68.5	31.5
G	1	0.67	0.0	2.5	3.1	0.0	2.5	0.0	44.5	55.5	0.0
	2	0.33	0.0	2.6	4.0	0.0	2.6	0.0	40.0	60.0	0.0
J	1	-0.50	0.0	0.0	8.5	0.0	0.0	0.0	0.0	100.0	0.0
	2	0.29	0.0	0.0	8.4	0.7	0.0	0.0	0.0	91.9	8.1
N	1	-0.14	0.0	0.0	7.6	0.0	0.0	0.0	0.0	100.0	0.0
	2	0.50	0.0	4.4	4.2	0.0	4.4	0.0	51.5	48.5	0.0
	3	-0.83	0.0	3.4	5.5	0.0	3.4	0.0	38.3	61.7	0.0
			**South channel transects**								
A	1	NA	32.1	154.0	229.9	0.0	186.1	7.7	37.0	55.3	0.0
B	1		0.0	96.9	225.0	0.0	96.9	0.0	30.1	69.9	0.0
D	1		0.0	0.0	0.0	0.0	0.0	0.0	0.0	100.0	0.0
E	1		0.0	0.0	0.0	0.0	0.0	0.0	0.0	100.0	0.0
H	1		0.0	14.7	286.6	0.0	14.7	0.0	4.9	95.1	0.0
I	1		0.0	0.0	0.0	0.0	0.0	0.0	0.0	100.0	0.0
L	1		0.0	44.6	262.5	0.0	44.6	0.0	14.5	85.5	0.0

Replicate (Rep) cross-channel observations were made at various stages of the tide. Along-channel transects were made within 1-2 h of slack high water.

**Figure 7:**
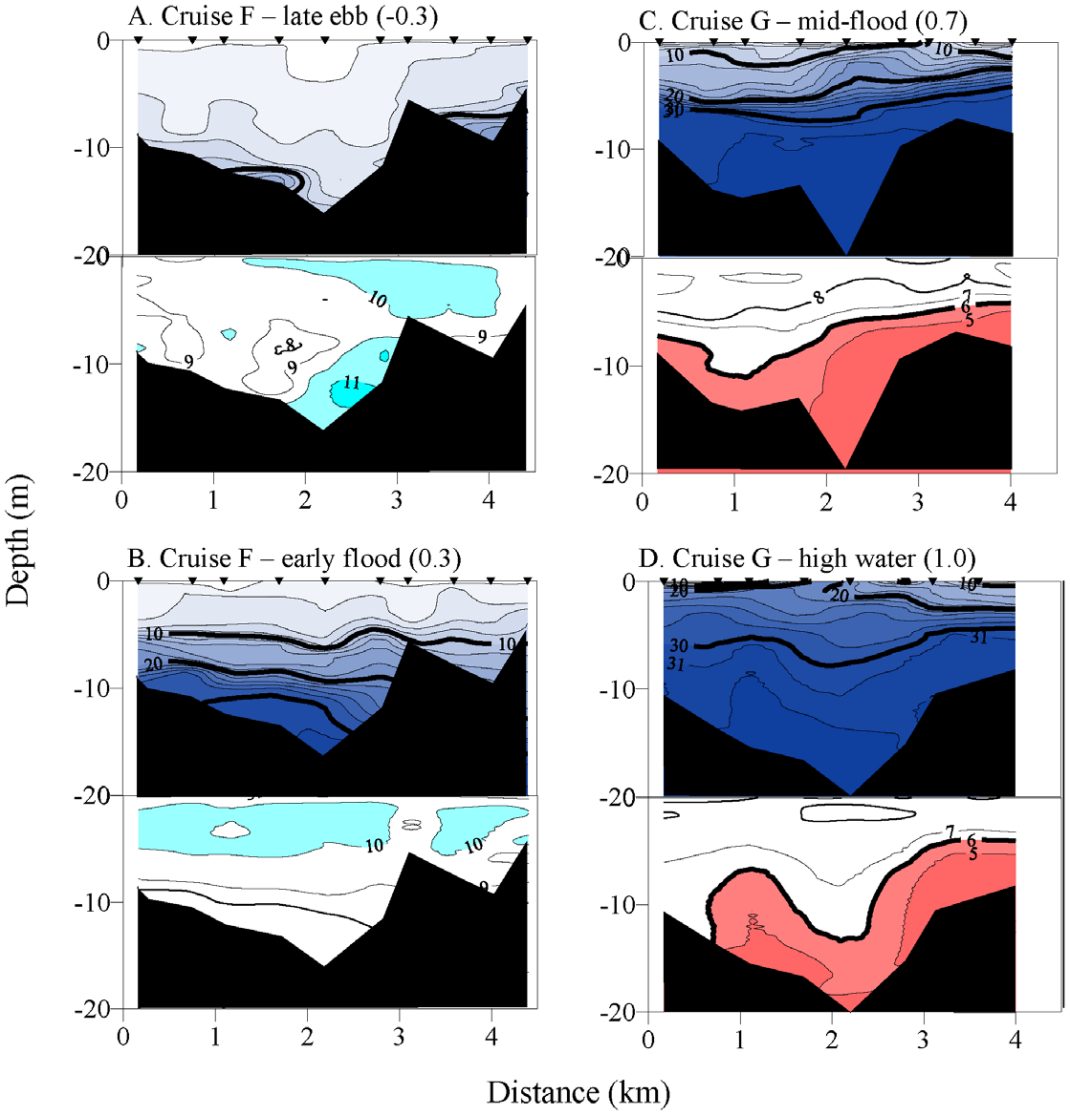
Across-channel transects of vertical water column salinity and dissolved oxygen concentration during May 2007. Salinity, top panels. Oxygen concentration, bottom panels. Plots A and B depict downwelling and C and D show upwelling conditions. For each date, two transects were made as indicated in the header. Isopleth and fill designations are as in Figure 6.

Two cruises in May 2007 were made a week apart, the first during low wind stress (Cruise F) and the second during strong upwelling conditions (Cruise G). Two measurements of the Mouth Line transect were made during each date (Figure 7). The entire water column was normoxic or supersaturated during low wind stress conditions (Figures 7A and B), while 40–45% of the water column (to 5 m) was under mild biological stress during upwelling (Figures 7C and D). Similarly, two cruises in August 2007 were made one week apart, the first during low wind stress (Cruise J) and the second during strong upwelling conditions (Cruise N), and similar results were found (Table 2). The three measurements of the Mouth Line transect that were made during Cruise N show the flushing of low DO water from the lower estuary during ebb tide (Table 2).

**Figure 8:**
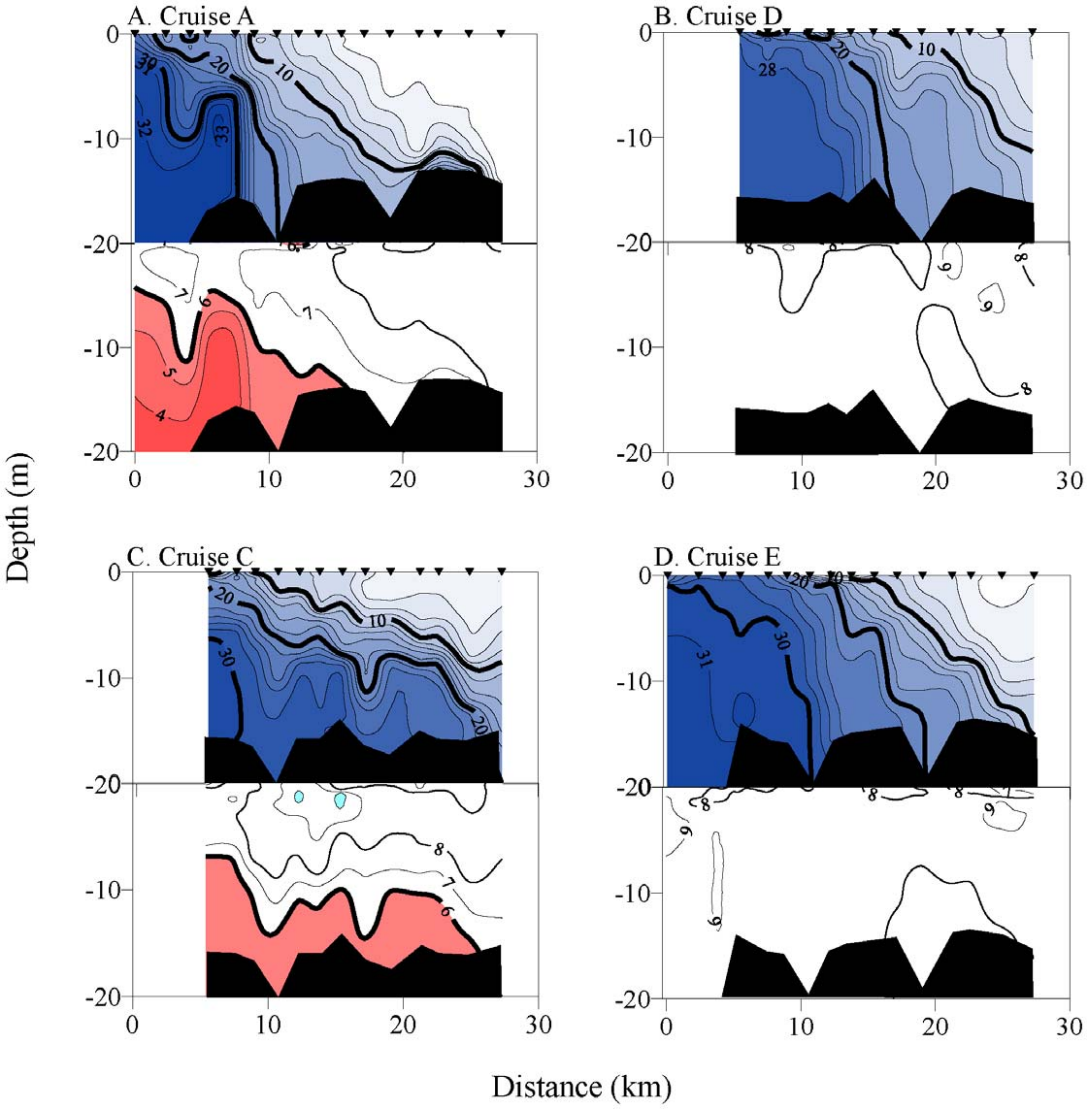
Along-channel channel transects of vertical water column salinity and dissolved oxygen concentration during Autumn 2006. Salinity, top panels. Oxygen concentration, bottom panels. Cruises shown in Plots A and C occurred during upwelling, while cruises in Plots B and D occurred during downwelling conditions. Isopleth and fill designations are as in Figure 6.

In the along-channel dimension, we sampled the South Channel transect on four dates during upwelling (Cruises A, C, H, L) and three dates during downwelling or low wind stress (Cruises D, E, I). Examples contrasting up- and downwelling conditions are shown for four surveys (A, C, D, E) in 2006 (Figure 8). These examples portray the typical salt wedge estuarine circulation observed in the Columbia estuary [26]. Biologically stressful DO levels were found only during upwelling periods, with up to 45% of the water column affected (Table 2). Low DO in bottom waters penetrated up to 25 km in the South Channel (Figure 8C). Thus, a large area of the estuarine water column could be affected by low DO events (up to 186×10^3^ m2 in the x-z dimension). The lowest DO values were invariably located in the highest salinity waters close to the estuary mouth.

Nine anchor station studies were conducted to investigate the tidal-scale temporal evolution of low DO events (Table 3): Cruise B occurred near NC14 in August 2006; two pairs of observations from NC14 and NC08 were conducted under neap tide (Cruises J and K) and spring tide (Cruises M and N) conditions in August 2007; four stations near SC09 (Cruises O–Q) or NC10 (Cruise R) were observed under contrasting spring/neap tidal patterns under strong upwelling during July 2008 (Figure 1; Table 1).

**Table 3:** Percent of time bottom waters were at various dissolved oxygen categories (mg L^−1^) during anchor station studies.

Cruise ID	Station	Study length	DO category		
			>2–4	>4–6	>6–10
B	NC07	4.7	17.0	53.2	29.8
J	NC07	8.4	0.0	0.0	100.0
K	NC14	9.0	0.0	0.0	100.0
M	NC14	7.6	0.0	55.3	44.7
N	NC07	7.8	17.9	61.5	20.5
O	SC09	7.9	100.0	0.0	0.0
P	SC09	4.5	100.0	0.0	0.0
Q	SC09	4.9	46.9	13.9	39.2
R	NC10	7.0	11.4	15.0	73.6

Study length in hours.

**Figure 9:**
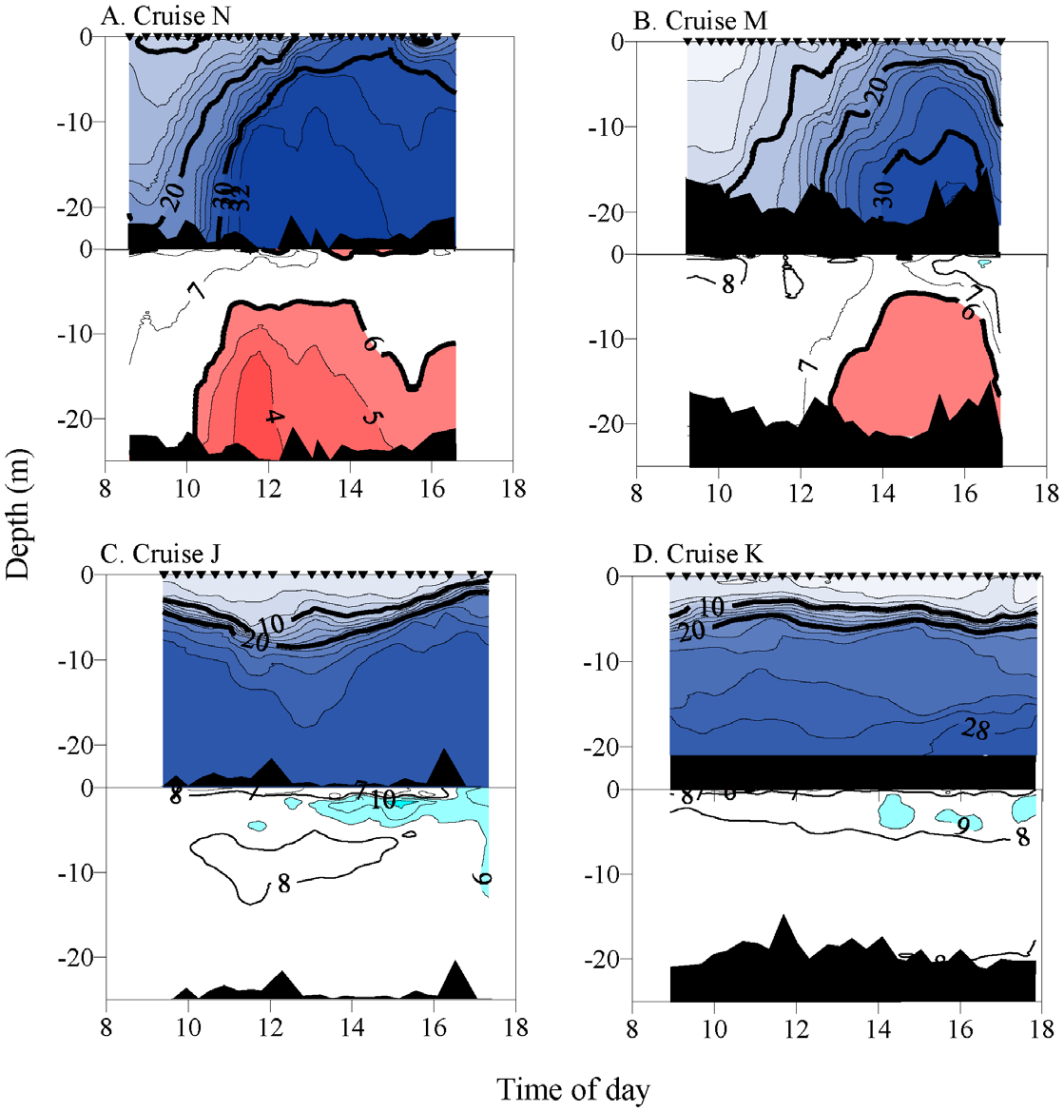
Anchor station time series of vertical water column salinity and dissolved oxygen concentration during August 2007. Salinity, top panels. Oxygen concentration, bottom panels. Plots A and B show upwelling during spring tides, which contrasts with Plots C and D that show downwelling during neap tides. Isopleth and fill designations are as in Figure 6.

In 2006 and 2007, spring tide sampling occurred during ebb-to-flood tide periods, when flooding water entered the estuary as a salt wedge. At NC14 near the estuary mouth, oxygen concentrations decreased from normoxic to <4.0 mg L^−1^ in a one hour period as ocean water intruded into the estuary (Figure 9A). Bottom oxygen conditions remained <6 mg L^−1^ for over 6.25 h (Table 3). Sampling the subsequent day at NC08, near the Astoria-Megler Bridge, revealed that low DO water was transported over 14 km upstream, where weakly stressful conditions at the bottom persisted for 3.9 h (Figure 9B, Table 3). At both stations, low DO conditions penetrated into the water column to within 5–8 m of the surface (60–70% of the water column), and S_max_ and O_2min_ occurred near high water. Similarly rapid onsets of stressful conditions were observed for other spring tide measurements. In contrast, during neap tides the water column was intensely stratified, and its structure varied little during the observation periods. However these neap tide samples (Cruises J and K) occurred during downwelling conditions, and no low DO water was detected (Figures 9C and D, Table 3).

**Figure 10:**
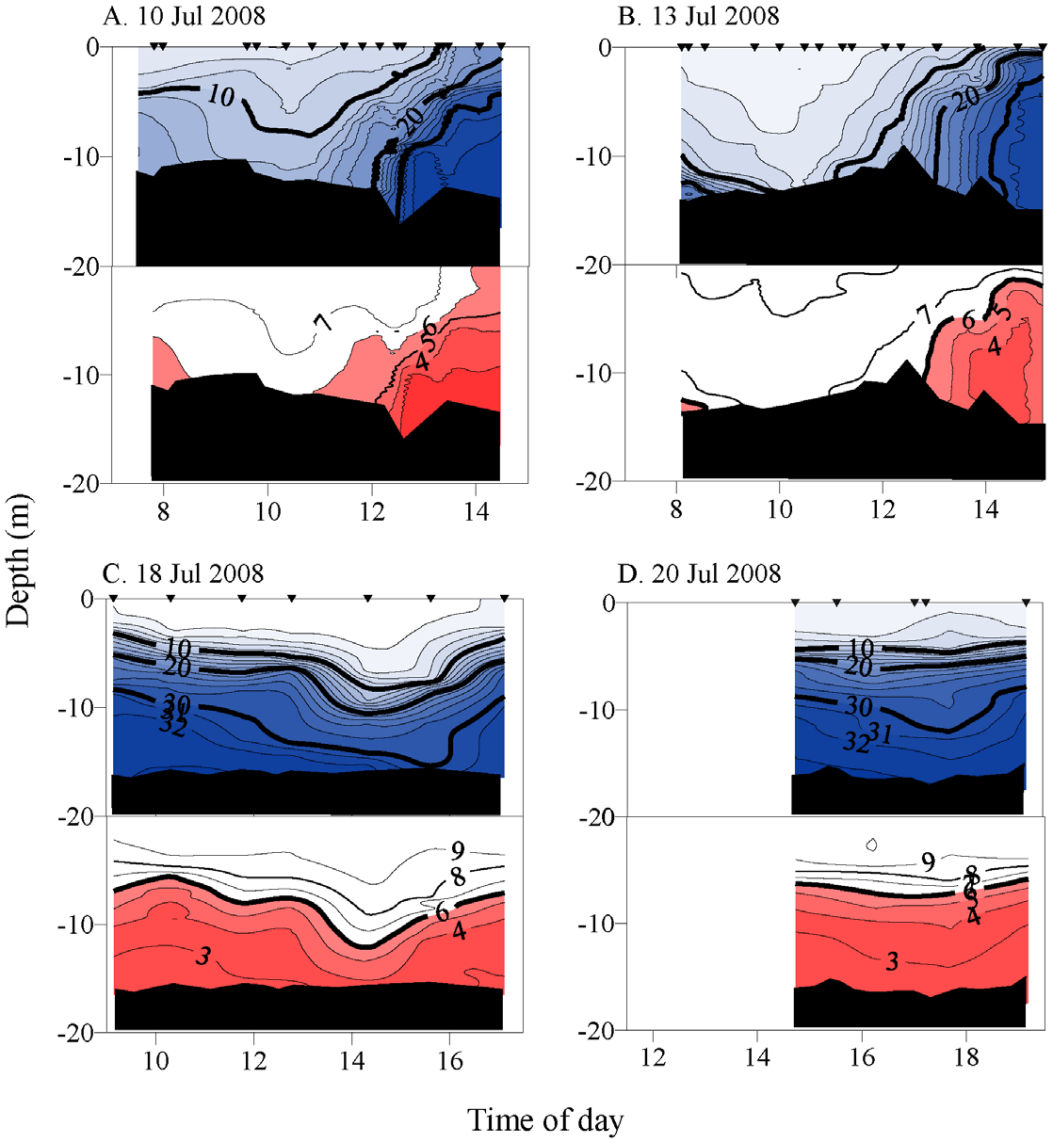
Anchor station time series of vertical water column salinity and dissolved oxygen concentration during July 2008. Salinity, top panels. Oxygen concentration, bottom panels. The plots contrast spring tides (A and B) from neap tides (C and D) during a strong upwelling event. Isopleth and fill designations are as in Figure 6.

In contrast, during 2008 there was a prolonged upwelling event during which we sampled both neap and spring tidal conditions. Moderately stressful to nearly hypoxic water was present in high salinity water during all four dates (Figure 10). During spring tides on 18 and 20 July, the water column structure again followed a salt wedge pattern as described above. Exposure to low DO occurred only when salinity exceeded 25–29 psu. At high tide, low DO water penetrated to within 2 m of the surface on 20 July and outcropped during 18 July (Figures 10A and B). Exposure to low DO conditions at the estuary benthic layer ranged from 26.4 to 60.8% of the measurement period (Table 3). In contrast, during the neap period of high stratification, moderately stressful to nearly hypoxic water was present in bottom water throughout the measurement periods (100% exposure), which were for 8 hours on 10 July and 4.5 h on 13 July (Figures 10C and D, Table 3). Mildly to moderately stressful levels were present within 5–6 m of the surface at high water, and encompassed 56.8 and 59.1%, respectively, of the space-time periods measured. These data show low DO water was associated with high salinity water, was maximal in vertical extent around high tide, and persisted or was advected in accordance with semidiurnal tidal patterns.

The annual number of upwelling events and the number of days per year estimated to have reduced DO in the estuary varied among years (Table 4). Based on the 4-d cumulative wind stress time series, there ranged an almost two-fold difference (6 and 11) in the number of low DO events between 2006 and 2008. The total number of days estimated to have DO <6 mg L^−1^ ranged from 103 to 152 (49 to 72% of the periods evaluated). Based on wind stress intensity, periods of severe low were rare, while periods of moderate DO stress ranged from 13 to 23% of the upwelling period. There was no relation between the number of low DO events and total number of days with stressful conditions, since the duration of the wind events varied. Thus, based on wind stress records, low DO events are likely to be common in the Columbia estuary.

**Table 4:** Number of upwelling events estimated from the wind stress time series, and the predicted duration of each DO category (mg L^−1^) computed for the typical upwelling season (day of year 90 to 300).

Year	Events	DOCategory	Days	% Time
2006	6	0 to 2	4	1.9
		>2 to 4	48	22.9
		>4 to 6	100	47.6
		0 to 6	152	72.4
2007	9	0 to 2	1	0.5
		>2 to 4	27	12.9
		>4 to 6	75	35.7
		0 to 6	103	49.0
2008	11	0 to 2	8	3.8
		>2 to 4	31	14.8
		>4 to 6	95	45.2
		0 to 6	134	63.8

Predictions were based on the regression equation O_2_min = 6.47× CWS +6.0.

## Discussion

### Origins of low DO water

Low DO occurs naturally in subsurface waters of the California Current System, but the origin of this source water can be from either the north or south of the Columbia estuary depending on the influence of large-scale climatic factors on the North Pacific Gyre [41]. Waters upwelled on the shelf appear to come primarily from the northward-flowing California Current Undercurrent at depths of 150–300 m. Feely et al. [42] found water from a depth of 150 m was upwelled to the surface from about 40 km offshore to the nearshore zone during strong upwelling. In addition to being oxygen-depleted, this water was also undersaturated with respect to aragonite and was therefore acidic to calcified organisms. In contrast, Wheeler et al. [20] and Grantham et al. [22] concluded the hypoxic mortalities that occurred on the Oregon shelf in 2002 were due to a combination of low DO source waters plus the oxidation of organic matter originating from the decline of a large diatom bloom that sank and was respired in the benthic bottom layer. In that case, the source waters were determined to be from an anomalous southward transport of subarctic water which was unusually high in nitrate and low in DO [20], [43]. Kaplan et al. [44] found interannual variation in the timing and intensity of upwelling led to variation in the source of upwelled water off Central California between 2006 and 2007. It appears both physical advection and biological processes affect the character of water intruding on the shelf, which then becomes the source water for upwelling, and several recent studies have documented low DO water on the Oregon shelf in proximity to estuarine systems [7], [19]–[22], [45]. Processes that supply oxygen-depleted water to the nearshore zone, whether the mechanism be variation in along-shelf advection, wind-induced changes in upwelling intensity, in situ depletion due to heterotrophic respiration, or some combination of these factors, would all contribute to a nearshore pool of low DO source water that can be advected into Pacific Northwest estuaries.

### Wind forcing

We sampled the distribution of DO in the Columbia estuary on 18 cruises that spanned a range of both salinities and of upwelling- and downwelling-favorable wind stresses. The results clearly show that low DO water was invariably associated with the ocean end-member, while the river end-member was consistently normoxic. Further, low DO events occurred during upwelling wind events, and when downwelling winds predominated, the water of maximum salinity was generally normoxic. Based on O_2min_, the intensity of low DO events was positively related to the strength of upwelling winds, indicating greater upwelling results in lower DO in the estuary. Transitions between up- and downwelling conditions, and the delivery of low DO water to the estuary, varied rapidly over the course of a few days. Our findings confirm the origin of low DO water in the Columbia estuary was upwelled subsurface water advected into the estuary from the ocean, and corroborate findings from the 1950s and 1960s as reported by Pearson and Holt [24] and Haertal et al. [25].

### Tidal forcing

The distribution, duration, and extent of low DO events varied temporally on semidiurnal tidal and spring/neap tidal scales which, along with streamflow, determine estuarine circulation patterns in the CRE. The density structure of the estuary modulates from a mainly vertically mixed, horizontally stratified (salt wedge) system during spring tides to a highly stratified state during neap tides [26], [40]. During spring tides, the estuary was well flushed on semidiurnal time scales, and high salinity, low DO water that intruded during flood tides was replaced by normoxic river water during the subsequent ebb flow (Figures 6B,7A). Exposure times of biologically stressful levels of oxygen to organisms during these periods were limited to a maximum of about 6 h near the estuary mouth and 4 h upstream near station NC14 (Table 2); however, the vertical extent of low DO was greatest during spring tides and could even outcrop to the surface (Figures 6A and 10A). During neap tides, estuarine waters were highly stratified, flushing was reduced, and high salinity bottom water had increased residence in the estuary (Table 3). For example, during the strong upwelling event in July 2008 (Figure 9), the neap tide series caused a much longer exposure of low DO to benthic organisms than during the following spring tide series when flushing was increased (100% exposure versus 36% for neap versus spring tide sampling periods of similar length). Stressful conditions at the benthic layer were maximized when strong upwelling occurred around neap tides. In contrast, the greatest vertical affect was found during spring tides at sections near the mouth of the estuary, when the entire water column could be rendered stressful. Together, time series and transect data indicated that large expanses of the water column and benthic layer were exposed to low DO water.

### Autochthonous oxygen production in the CRE

Despite the highly advective nature of the Columbia estuary [40], biological activity by phototrophic organisms was a source of oxygen within the estuary. Surface waters were frequently supersaturated in water of mesohaline salinity (Figures 2–4, Cruises C–E, I–L). This indicates an autochthonous estuarine source of oxygen. During summer and early autumn, the chlorophyll signal in the Columbia estuary is usually dominated by the highly motile mixotrophic ciliate *Myrionecta rubra*, which forms dense “red water” blooms [26], [46]. The distribution of *M. rubra* was spatially coincident with high oxygen concentrations during August and September [26] and usually occurred at salinities between 3 and 20 psu. The highest vertical oxygen gradients (−1.3 mg L^−1^ m^−1^) were found in shallow water during autumn, when low DO ocean water intruded under this estuarine-produced supersaturated surface waters (Figure 6C). However, the biological impact of biota was also found during May 2006, when 38% of the water column was supersaturated along the Mouth Line transect (Figure 7A–B). This production was likely due to riverine diatoms [25]. Wheeler et al. [20] found supersaturated surface conditions within 10 km of the Oregon coast during July 2002, but this occurred in salinities >33 psu and is indicative of production by coastal phytoplankton. At present, it is not well determined how oxygen production by phytoplankton may alleviate low DO in upwelled water; however the same upwelled water that is low in DO is high in nitrate and therefore is stimulatory for phytoplankton growth. The normoxic oxygen levels observed in the ocean end-member during downwelling conditions may in part be due to oxygen production by coastal phytoplankton and the subsequent transport of those water masses to the estuary [19].

### Potential effects of low DO on salmon and crab

Each year during the upwelling season, around 100 million juvenile Pacific salmon migrate through the Columbia estuary to the ocean. There are no field measurements to discern the impacts of low DO on these salmon; however, information from the literature suggests there are effects and interactions that would reduce fitness and/or increase predation. These include avoidance behavior and subsequent habitat constriction, impaired swimming ability, and reduced feeding and growth [14]. Studies have demonstrated that salmon are relatively intolerant to low DO and usually exhibit avoidance behaviors when exposed to reduced oxygen levels. A review by Davis [11] concluded the average minimum incipient response threshold for salmonids in freshwater was 6.0 mg L^−1^, while the US Environmental Protection Agency (EPA) [14] rated 3.0 mg L^−1^ as the limit to avoid acute mortality. Whitmore et al. [47] found juvenile Chinook (*O. tshawytscha)* and coho (*O. kisutch*) salmon avoided water <4.5 and 6.0 mg L^−1^, respectively, and both species preferred concentrations >9.0 mg L^−1^. Birtwell and Kruzynski [48] found that juvenile Chinook salmon avoided low DO bottom water in the highly stratified Somass River estuary, BC, Canada, and, by this avoidance behavior, the salmon became isolated in unsuitably warm and polluted surface areas with reduced prey resources. In laboratory experiments, these authors found surface-oriented Chinook salmon were induced to descend into saline water when oxygen concentrations in the surface layer were decreased from 9 to between 7 and 8 mg L^−1^. It should be noted that returning adult salmon also avoid water with oxygen concentrations below about 3.5 mg L^−1^ [49], [50]. Many other fish and invertebrate species undergo vertical and horizontal habitat displacements to avoid intruding low DO bottom water (e.g., [9], [51], [52]).

Migrating salmon have been shown to exhibit a subsurface preference [53]–[55], and a vertical range restriction could have profound impacts on salmon migrating through the Columbia estuary. This is due to the high densities of piscivorous birds such as Caspian terns (*Hydroprogne caspia*) that nest and roost on East Sand Island in the lower estuary [56]. Price and Schreck [57] and Kennedy et al. [58] suggest avoidance of high salinity water due to stress or maladaptation of osmoregulation may concentrate migrating fish to low salinity surface layers, where they maybe more vulnerable to predation by shallow-feeding plunge-divers. An avoidance response to low DO in intruding ocean water could similarly increase predation rates on salmon by these birds. DO levels during our study were commonly <6.0 mg L^−1^ within 5 m of the surface and low DO water occasionally outcropped at the surface at levels that likely induced a behavioral response by salmon. Moreover, low DO progressively reduces swimming speed in juvenile salmon (by approximately 20% at 4.0 mg L^−1^ [59]–[61], with the consequence that escape responses to diving birds such as double-crested cormorants (*Phalacrocorax auritus*) or predatory fishes could be impeded. Note that the two bird species mentioned here are estimated to consume millions of migrating salmon annually [62], [63]. We speculate periods of shoaling low DO water in the Columbia estuary may lead to increased predation rates by birds on salmon as well as on other small pelagic fishes.

Salmon likely suffer additional non-lethal effects to low DO exposure. Summarizing a series of growth experiments with juvenile Chinook and coho salmon in freshwater, the U.S. EPA [14] reported exponential decreases in median growth from a minimal effect at 7.0 mg L^−1^ to a decrease of 42±7.1% at 3.0 mg L^−1^. Growth reductions for salmon exposed to DO levels below 4.0 mg L^−1^ were considered to be severe. The critical criterion for growth of saltwater organisms was determined to be 4.8 mg L^−1^ [38]. The mechanism for growth reductions with increasing oxygen stress appears to be a combination of enhanced metabolic demand and decreased feeding. Moreover, stress has been found to increase predation rates on juvenile salmonids [64], [65], which suggests that salmon metabolically weakened or stressed by low DO events may be more susceptible to predators. Low DO in surface waters on the shelf may also affect salmon once they migrate from the estuary [e.g., 46]. Thus, while DO levels in the Columbia estuary are unlikely to be directly lethal to salmonids, behavioral and physiological responses to low DO that reduce performance and increase stress may increase predation risk and/or decrease fitness of migrating fish. More research is required to elucidate behavioral and sublethal effects of low DO on salmon.

Pacific Northwest estuaries such as the Columbia are also important nursery areas for Dungeness crabs [66], and benthic fauna with limited mobility are often considered more susceptible to low DO events than mobile nekton. Shelf anoxia in 2002 led to widespread mortality of benthic organisms, including Dungeness crab [22], but crabs are also affected by non-lethal oxygen concentrations. Bernatis et al. [67] determined Dungeness crabs to be relatively intolerant of DO levels <47% saturation (which is more tolerant than most crustaceans, [11]), and Stone and O'Clair [68] similarly found adult crabs in Barkley Sound avoided water <50% saturation. Crabs reduce their food intake in hypoxia, and while they may forage in low DO areas, they tend to move to areas of higher DO concentration for digestion (which consumes oxygen). One point of concern regards the cumulative effects of low DO and salinity on Dungeness crab biology in the Columbia estuary. *Cancer magister* is a weak osmoregulator and becomes inactive at reduced salinities [69]. Sugerman et al. [70] found crabs reduce pumping water over the gills to curtail ionic loss at 23 psu, and cease pumping at 16 psu. Curtis et al. [71] found crabs reduced both overall feeding activity and the quantity of food ingested at mesohaline salinities. Salinity levels in the Columbia estuary commonly fall below 16 psu during ebbing tides (e.g., Figures 7, 10), at which time crabs are probably quiescent. If activity during subsequent flood tide is also limited due to low DO, then the time available for crab foraging could be reduced, as was found for blue crab (*Callinectes sapidus*) by Seitz et al. [72]. Further uncertainties include the effects of low DO on settlement and survival of *C. magister* megalopae (the recruiting larval form), which usually enter Pacific Northwest estuaries from April through November [73]. For megalopae of the Atlantic rock crab (*C. irroratus*), the median lethal concentration (LC_50_) values during 240 min tests ranged from 1.2 mg L^−1^ at 10°C to 3.3 mg L^−1^ at 30°C [74], which suggests larval crab could be affected by DO values we recorded in the Columbia estuary. It is not known if low DO affects metamorphic success of larval Dungeness crab.

### Potential effects of climate change

Although low DO in the coastal Pacific Northwest is not a new phenomenon [24], [25], long-term trends indicate the frequency and severity of low DO events may be increasing due to climatic alterations in atmospheric forcings and changes in ocean circulation patterns [23], [75]. The depth of the oxygen minimum zone in the California Current System has been shoaling [23], [75]–[77], and this is the source water for coastal upwelling on the Pacific Northwest continental shelf. Modeling predicts further declines in DO are likely to occur, based on increased isolation of bottom waters by strengthened thermal stratification and reduced vertical mixing [78]. However, the most recent review of coastal estuaries in the Pacific Northwest failed to identify low DO as problematic [79], and the impact of low DO on organisms in the Columbia estuary and other PNW estuaries has not been ascertained. Based on a wind-forced supply mechanism, low DO events in the Columbia estuary appear to be common at present (Table 4), and along with increased acidification of this subsurface water [42], [80], shelf and estuarine habitats could be experiencing declines in environmental quality.

### Summary

1. Low DO water was advected into the Columbia River estuary from the ocean end-member; the riverine end-member was always normoxic.

2. Oxygen depleted water in the estuary was associated with upwelling wind events, and low DO was not detected during periods of downwelling or low wind stress.

3. Spring-neap tidal stratification largely determined levels of exposure to low DO water, with greater vertical extent during spring tides and greater horizontal and benthic impacts during neap tides.

4. Hypoxic oxygen concentrations were not measured during our sampling; however, DO levels were sufficiently low to affect the behavior of migrating juvenile salmon and benthic crabs (according to literature values).
